# Draft Genome Sequence of Enterobacter hormaechei Strain MHSD6, a Plant Endophyte Isolated from Medicinal Plant *Pellaea calomelanos*

**DOI:** 10.1128/MRA.01251-19

**Published:** 2019-11-27

**Authors:** Khuthadzo Tshishonga, Mahloro H. Serepa-Dlamini

**Affiliations:** a Department of Biotechnology and Food Technology, University of Johannesburg, Johannesburg, South Africa; University of Maryland School of Medicine

## Abstract

We describe here the draft genome sequence of Enterobacter hormaechei strain MHSD6, a bacterial endophyte isolated from the medicinal plant Pellaea calomelanos. The Enterobacter hormaechei strain MHSD6 draft genome is 4,817,102 bp in length, with a G+C content of 55.50%.

## ANNOUNCEMENT

Endophytes are a group of microorganisms, mostly bacteria and fungi, which colonize internal plant tissues without causing any harm (1). Most plant species host a variety of endophytes, and therefore novel endophytic microorganisms exist within plant species growing in unique environments (2). Endophytes have a symbiotic relationship with their host plants. Plants provide the endophyte with carbon for energy, as well as with an environment for growth and survival, and in turn, endophytes function as biological defense for the plant through production of secondary metabolites with toxicity against infected host cells and phytopathogens (2, 3). In addition, endophytes promote plant growth through production of phytohormones and nitrogen fixation, as well as by solubilization of minerals such as phosphorus (4). Endophytes can produce the same or similar secondary metabolites as their plant hosts; thus, endophytes associated with medicinal plants offer alternative sources for isolation of bioactive compounds (4). Endophyte genomes will enhance understanding of their symbiotic relationships with plants and further reveal information on how their genomic profiles differ from those of clinical or pathogenic strains.

Enterobacter hormaechei strain MHSD6 was isolated from sterilized leaves of the medicinal plant Pellaea calomelanos, which was obtained from Botlokwa (23°2934.8′S, 29°4211.2′E) in Limpopo Province, South Africa. The bacterial endophyte was isolated from sterilized leaves and cultivated as described by Mahlangu and Serepa-Dlamini (5). Briefly, following serial sterilization with 70% ethanol and 1% sodium hypochlorite, the plant leaves were ground in 2 ml of saline, and the homogenate was streaked onto nutrient agar plates, followed by incubation at 28°C for 48 h. The chromosomal genome was extracted from solid bacterial colonies using a NucleoSpin microbial DNA extraction kit following the manufacturer’s protocol. The concentration and quality of isolated DNA were determined using a NanoDrop ND-2000 UV-visible (UV-Vis) spectrophotometer. Paired-end libraries were generated using a NEBNext Ultra II DNA kit (Illumina) and sequenced with a paired-end sequencing strategy (2 × 300 bp) using an Illumina MiSeq instrument v3 at a commercial service provider (Agricultural Research Council [ARC] Biotechnology Platform, Pretoria, South Africa).

Quality control and assembly of the raw sequence reads were performed on the Galaxy web platform (https://usegalaxy.org) (6). FastQC v0.69 was used to assess the quality of the raw reads (7). The sequence reads were *de novo* assembled with Unicycler v0.4.1.1 (8) and assessed with Quast v4.6.3 (9), using default parameters. The complete genome sequence was submitted to NCBI and annotated using Prokaryotic Genome Annotation Pipeline (PGAP) (10) and the Rapid Annotations using Subsystems Technology (RAST) server (11–13). The genomic islands were identified by screening the PGAP annotation file generated from NCBI on the IslandViewer 4 website (http://www.pathogenomics.sfu.ca/islandviewer/) (14). Clustered regularly interspaced short palindromic repeats (CRISPR) were predicted by CRISPRCasFinder software (15–17).

The Illumina MiSeq platform generated 250 Mb of sequence reads. The draft genome of strain MHSD6 produced 60 contigs with a total genomic length of 4,817,102 bp and a G+C content of 55.50% (Table 1). It consists of a total of 4,779 genes; 4,691 of the genes are protein coding genes, 88 are RNA genes, 143 are pseudogenes, and 6 are noncoding RNA (ncRNA) genes. The RNA coding genes predicted include 76 tRNA and 6 rRNA (5S, 16S, and 23S) genes. We identified genes responsible for nitrogen fixation, phytohormone production, transport proteins, and transcriptional regulators, all of which may play a part in symbiosis with the plant and promote plant growth.

**TABLE 1:** Genome data of Enterobacter hormaechei strain MHSD6

Attribute	Value
Genome size (bp)	4,817,102
G+C content (%)	55.50
Total no. of genes	4,779
No. of protein coding genes	4,691
No. of RNAs	88
No. of rRNA genes	6
No. of tRNA genes	76
No. of genomic islands	33
No. of CRISPRs	2

### Data availability.

This whole-genome shotgun project and associated data have been deposited at DDBJ/ENA/GenBank under the accession number VHQJ00000000, BioProject accession number PRJNA550302, BioSample accession number SAMN12124847, and SRA accession number SRR10237366. The version described in this paper is version VHQJ02000000.
